# Oral administration of lysozyme protects against injury of ileum via modulating gut microbiota dysbiosis after severe traumatic brain injury

**DOI:** 10.3389/fcimb.2024.1304218

**Published:** 2024-01-30

**Authors:** Weijian Yang, Caihua Xi, Haijun Yao, Qiang Yuan, Jun Zhang, Qifang Chen, Gang Wu, Jin Hu

**Affiliations:** ^1^ Department of Neurosurgery, Huashan Hospital, Shanghai Medical College, Fudan University, Shanghai, China; ^2^ National Center for Neurological Disorders, Shanghai, China; ^3^ Shanghai Key Laboratory of Brain Function Restoration and Neural Regeneration, Shanghai, China; ^4^ Neurosurgical Institute of Fudan University, Shanghai, China; ^5^ Shanghai Clinical Medical Center of Neurosurgery, Shanghai, China; ^6^ Department of Neurosurgery and Neurocritical Care, Huashan Hospital, Shanghai Medical College, Fudan University, Shanghai, China

**Keywords:** traumatic brain injury, gut microbiota, Paneth cell, lysozyme, bacterial translocation

## Abstract

**Objective:**

The current study sought to clarify the role of lysozyme-regulated gut microbiota and explored the potential therapeutic effects of lysozyme on ileum injury induced by severe traumatic brain injury (sTBI) and bacterial pneumonia *in vivo* and *in vitro* experiments.

**Methods:**

Male 6–8-week-old specific pathogen-free (SPF) C57BL/6 mice were randomly divided into Normal group (N), Sham group (S), sTBI group (T), sTBI + or Lysozyme-treated group (L), Normal + Lysozyme group (NL) and Sham group + Lysozyme group (SL). At the day 7 after establishment of the model, mice were anesthetized and the samples were collected. The microbiota in lungs and fresh contents of the ileocecum were analyzed. Lungs and distal ileum were used to detect the degree of injury. The number of Paneth cells and the expression level of lysozyme were assessed. The bacterial translocation was determined. Intestinal organoids culture and co-coculture system was used to test whether lysozyme remodels the intestinal barrier through the gut microbiota.

**Results:**

After oral administration of lysozyme, the intestinal microbiota is rebalanced, the composition of lung microbiota is restored, and translocation of intestinal bacteria is mitigated. Lysozyme administration reinstates lysozyme expression in Paneth cells, thereby reducing intestinal permeability, pathological score, apoptosis rate, and inflammation levels. The gut microbiota, including *Oscillospira*, *Ruminococcus*, *Alistipes*, *Butyricicoccus*, and *Lactobacillus*, play a crucial role in regulating and improving intestinal barrier damage and modulating Paneth cells in lysozyme-treated mice. A co-culture system comprising intestinal organoids and brain-derived proteins (BP), which demonstrated that the BP effectively downregulated the expression of lysozyme in intestinal organoids. However, supplementation of lysozyme to this co-culture system failed to restore its expression in intestinal organoids.

**Conclusion:**

The present study unveiled a virtuous cycle whereby oral administration of lysozyme restores Paneth cell’s function, mitigates intestinal injury and bacterial translocation through the remodeling of gut microbiota.

## 1 Introduction

The global incidence of neurotrauma is estimated to exceed 27 million annually, with severe traumatic brain injury (sTBI) continuing to demonstrate high mortality rates (20-40%) and significant morbidity among survivors (Lazaridis and Foreman, 2023). The prognosis of patients with sTBI is often compromised by the presence of bacterial pneumonia, which significantly impacts their overall outcome (Ziaka and Exadaktylos, 2021). The gut-lung axis is gaining increasing attention as research progresses, and it has been postulated that pulmonary lesions may originate from the gastrointestinal tract (Mjosberg and Rao, 2018). In addition, the gastrointestinal tract serves as a significant reservoir for multidrug-resistant bacteria (Corriero et al., 2022). Clinical study has demonstrated that *Enterobacteriaceae* predominantly constitute the bacterial population responsible for lung infections in patients with brain injuries (Yang et al., 2023). Furthermore, TBI often coexists with intestinal barrier dysfunction and dysbiosis, thereby creating opportunities for the translocation of gut bacteria (Yang et al., 2022). Therefore, the gut microbiota plays a crucial role in the progression of pulmonary inflammation and infection after TBI.

Lysozyme is highly esteemed for their capacity to cleave the β-(1,4)-glycosidic bonds within peptidoglycan (Beaussart et al., 2021). The well-established role of hydrolytic action in antimicrobial defense lies in its capacity to disrupt the structural integrity of the bacterial cell wall (Nawaz et al., 2022). The primary source of lysozyme within the small intestine is derived from Paneth cells, which serve a critical function in maintaining intestinal microbiota homeostasis (Cray et al., 2021). The expression of lysozyme in Paneth cells was found to be reduced after sTBI, leading to an increased bacterial translocation to the lung (Yang et al., 2022). Furthermore, a correlation was observed between the decreased levels of lysozyme and gut bacterial translocation (Yang et al., 2022).

In this study, we investigated the role of lysozyme-regulated gut microbiota and explored the potential therapeutic effects of lysozyme on ileum injury induced by sTBI and bacterial pneumonia through *in vivo* and *in vitro* experiments.

## 2 Materials and methods

### 2.1 Mice and modeling

#### 2.1.1 Mice and housing

Male 6–8-week-old specific pathogen-free (SPF) C57BL/6 mice were purchased from Shanghai SLAC Laboratory Animal Corporation Limited and housed under SPF conditions. The mice were acclimatized for one week under temperature-controlled conditions (23 ± 2°C), maintaining a 12 h/12 h light/dark cycles and allowing mice to freely harvest energy. The provision of sterilized drinking water was accompanied by the distribution of clean bottles, which were washed daily. Additionally, the cages and pads were replaced every three days. This study was approved by the ethics committee of Huashan Hospital Affiliated to Fudan University (2022JS Huashan Hospital-319, 25 February 2022). All experimental procedures and animal welfare standards strictly followed the guidelines of animal experimental research report (Percie et al., 2020).

#### 2.1.2 Animal model

Mice were randomly divided into 6 groups (n = 5): Normal group (N), Sham group (S), sTBI group (T), sTBI + Lysozyme group (L), Normal + Lysozyme group (NL) and Sham group + Lysozyme group (SL). In T and L group, mice were anesthetized by intraperitoneal injection of 0.6% sodium pentobarbital. The bone window was located 1 mm behind the coronal suture and 3 mm on the left side of the sagittal suture. The bone window was drilled with a 4 mm diameter dental drill to expose the complete dura. The sTBI model was established by Pinpoint PCI 3000 system (Hatteras Instruments Inc., U.S.A). The parameters were set as follows (Yang et al., 2022): the diameter of the striking head was 3 mm, the depth was 3 mm, the strike speed was 1.5 m/s, and the strike time was 100 ms. The mice in S group received anesthesia and craniotomy. The mice in the L, NL and SL group received an oral gavage of 200 U/day lysozyme (Sigma-Aldrich, USA) for 1 week after modeling (Fu et al., 2022). Mice in the N, S and T group were received an equal volume of sterilized drinking water.

#### 2.1.3 Sample collection

At the day 7 after establishment of the model, mice were anesthetized with sodium pentobarbital and euthanized by exsanguination. Blood samples, distal ileum, and lungs were collected and stored at -80°C or in 4% paraformaldehyde. Fresh contents of the ileocecum were collected and stored at -80°C for analysis of the gut microbiota. The right lung was stored at -80°C until sequencing. The left lungs were prepared for histochemical protein examination.

### 2.2 Histological analysis

#### 2.2.1 Hematoxylin and eosin (HE) staining

Fresh distal ileum and lungs were soaked in 4% paraformaldehyde and dehydrated. The tissues were embedded in paraffin, and sectioned at 5μm. Tissues were dewaxed, rehydrated, and stained. Morphological analysis of lung and distal ileum sections were stained with HE (Beyotime HE Staining Kit, China) following manufacturer recommendations. Histopathological injuries were examined by using a light microscope (Olympus Corporation, Japan). Lung injury was assessed according to the scoring criteria reported previously (Daniel et al., 2021). In brief, lung injury was analyzed by a blinded participant using a 4-point scale as follows: No detectable inflammation - 0, bronchioles surrounded by a few inflammatory cells – 1, bronchioles surrounded by a layer one cell deep – 2, bronchioles surrounded by a layer 2–4 cells deep – 3, bronchioles surrounded by a layer more than four cells deep – 4. The injury of the distal ileum was assessed with a modified version of the Chiu’s score (Huang et al., 2022): 0, normal villus and gland; 1, formation of Gruenhagen’s antrum at the top of the villus; 2, expansion of subepidermal Gruenhagen’s antrum and slightly injured gland; 3, enlargement of the subepidermal gap and engorgement of the capillary vessel; 4, epidermis moderately isolated with the lamina propria and injured gland; 5, top villus shedding; 6, obvious villus shedding and capillary vessel dilating; 7, lamina propria villus shedding and distinct injured gland; 8, initially decomposed lamina propria; 9, hemorrhage and ulceration. A minimum of five randomly selected areas from each specimen were evaluated and averaged to determine the extent of mucosal damage.

#### 2.2.2 Terminal deoxynucleotidyl transferase dUTP nick-end labeling (TUNEL) assay and immunofluorescence staining

Distal ileum apoptosis was evaluated by a TUNEL assay using a *In Situ* Cell Death Detection Kit (Roche, Germany). Slides were heated at 60°C for 1 h. Next, sections were dewaxed, rehydrated, and stained. For TUNEL assay, the staining methods according to the manufacturer’s instructions. The nuclei were counterstained with 4’,6-diamidino-2-phenylindole (DAPI) (Beyotime, China) at 4°C for 10 min. For quantification, we calculated the percentage of positive expression cells in specific intestine field using ImageJ (1.8.0_345, USA). Expression of lysozyme and zonula occludens-1 (ZO-1) in distal ileum tissues were detected by immunofluorescence staining. Briefly, the sections were incubated with anti-lysozyme (Abcam, USA) or anti-ZO-1 (Invitrogen, USA), respectively, overnight at 4°C. They were then incubated with Goat Anti-Rabbit IgG H&L secondary antibody (Abcam, USA). The nuclei were counterstained with DAPI at 4°C for 10 min. The expression levels of lysozyme of intestinal organoids were also detected by immunofluorescence staining. Images were captured with C2+ confocal microscope (Nikon, Japan). For quantification, we calculated the percentage of positive expression area using ImageJ (1.8.0_345, USA) and represented by the average optical density (AOD) value (AOD = IOD/area, IOD: integrated option density).

### 2.3 Myeloperoxidase (MPO) assay and enzyme-linked immunosorbent assay (ELISA)

#### 2.3.1 MPO assay

After homogenizing the lung tissue, activities of MPO in lung tissues were detected according to the instructions of MPO Activity Colorimetric Assay Kit (BioVision Incorporated, USA). In BioVision’s MPO Assay Kit, HClO produced from H_2_O_2_ and Cl^-^ reacts with taurine to generate taurine chloramine, which subsequently reacts with the trinitrobenzene probe to eliminate color (λ = 412 nm).

#### 2.3.2 ELISA

Blood samples were collected from the mouse heart by cardiac puncture into an EDTA-rinsed syringe. Plasma was obtained by centrifuging the blood with 1500 g for 15 min at 4°C. Samples were stored at -80°C until use. Plasma lipopolysaccharide binding protein (LBP) (Abcam, USA), soluble form CD14 (sCD14) (R&D Systems, USA) and Zonulin (MyBioSource, USA) were quantified by ELISA according to the manufacturer’s instructions. The levels of interleukin (IL) 1β, IL-6, and tumor necrosis factor (TNF) α in the ileum were quantified using ELISA kits (R&D Systems, USA) following the manufacturer’s instructions.

### 2.4 Intestinal organoids culture and co-coculture system

Intestinal organoids were obtained from C57BL/6 mice. We cultured the intestinal organoids in strict accordance with the protocol provided by Stemcell (https://www.stemcell.com/technical-resources/area-of-interest/organoid-research/intestinal-research/tech-tips-protocols/intestinal-epithelial-organoid-culture-with-intesticult-organoid-growth-medium-mouse-lp.html#protocols) (accessed on 17 December 2023). According to protocol, organoid culture is divided into 2 parts, namely: isolation of mouse intestinal crypts and organoids culture from crypts. Brain-derived proteins (BP) were obtained by repeated freezing and thawing. Under sterile conditions, mice brain tissue was granulated into single cell suspensions and was thoroughly mixed with 5 ml dulbecco’s phosphate-buffered saline (D-PBS) (Beyotime, China). This solution was centrifuged for 5 min at 1000 rpm/min, and the upper liquid was discarded and mixed with 2 ml D-PBS. The solution was repeatedly frozen and thawed by liquid nitrogen and room temperature, and the cell rupture was demonstrated by microscopy. A total of 100 ng BP and 200 U lysozyme were added to intestinal organoids in each well, and the mixture was incubated for 48h.

### 2.5 Western blotting

The expression levels of lysozyme in distal ileum and intestinal organoids were detected by Western blotting. The distal ileum and intestinal organoids were treated with radioimmunoprecipitation assay (RIPA) Lysis Buffer (Beyotime, China) and lysed. The lysates (50μg of protein) were separated by electrophoresis 12% Sodium Dodecyl Sulfate (SDS) polyacrylamide gels and transferred to polyvinylidene difluoride (PVDF) membranes (Millipore, USA). The membranes were incubated with Tris-buffered saline (TBS) containing 1% (w/v) nonfat-milk and 0.1% (v/v) Tween-20 (TBST) for 2 h to block non-specific binding, washed with TBST for 45 min, incubated with the anti-lysozyme (Abcam, USA) or β-actin (Abcam, USA) primary antibody at 4°C overnight, washed with TBST for 45 min, incubated with second antibody for 1 h, developed using enhanced chemiluminescence (ECL) (Thermo Fisher, USA). The expression level of ZO-1 (Anti-ZO-1 primary antibody, Invitrogen, USA) in the distal ileum was detected by the same method as above.

### 2.6 The 16S rRNA sequencing and microbiome analysis

16S ribosomal RNA (rRNA) sequencing was performed to classify bacterial DNA, which was isolated from the contents of the ileocecum and lung tissues microbiome. Quantitative polymerase chain reaction (PCR) of the bacterial 16S rRNA gene V3-V4 region was performed using the forward primer F (5′-ACTCCTACGGGAGGCAGCA-3′) and the reverse primer R (5′-GGACTACHVGGGTWTCTAAT-3′). The data from the 16S rRNA gene sequence were analyzed using QIIME 2 (2019.4) and R (v3.2.0) software packages. SourceTracker, popularly used in tracing the origin of bacteria, applies a Bayesian approach to determine the relative contributions of one or more designated “sources” to a particular “sink” microbiota (Knights et al., 2011). We employed SourceTracker to assess the contributions to the lung microbiota. The 16S rRNA sequencing and microbiome analysis was supported by Shanghai Personal Biotechnology Co., Ltd. (China).

### 2.7 Statistical analysis methods

All experiments were repeated at least 5 times, which made the study reproducible. All data are expressed as mean ± SD. Comparisons between groups were tested by one-way analysis of variance (Tukey’s test). The Divisive Amplicon Denoising Algorithm 2 (DADA2) was used to dereplication and produced the amplicon sequence variants. Alpha diversity was analyzed by Kruskal-Wallis test. The difference between different groups was analyzed based on the nonmetric multidimensional scaling (NMDS) with Anosim. Random forest analysis is an ensemble method (combination of multiple classifiers) based on generating a set of uncorrelated decision trees to make a prediction, making it robust and suitable for complex data patterns (Neri-Rosario et al., 2023). We used random forest as a machine-learning method to predicting the landmark bacteria. Spearman rank correlation coefficient to analyze the correlation between gut microbiota and indicators of ileal injury. SPSS 23.0 software was used to complete the statistical test. *P* < 0.05 was considered significantly different. The visualization figures were produced by genescloud (https://www.genescloud.cn) (accessed on 17 December 2023).

## 3 Results

### 3.1 Oral administration of lysozyme alleviates gut dysbiosis

**Figure 1:**
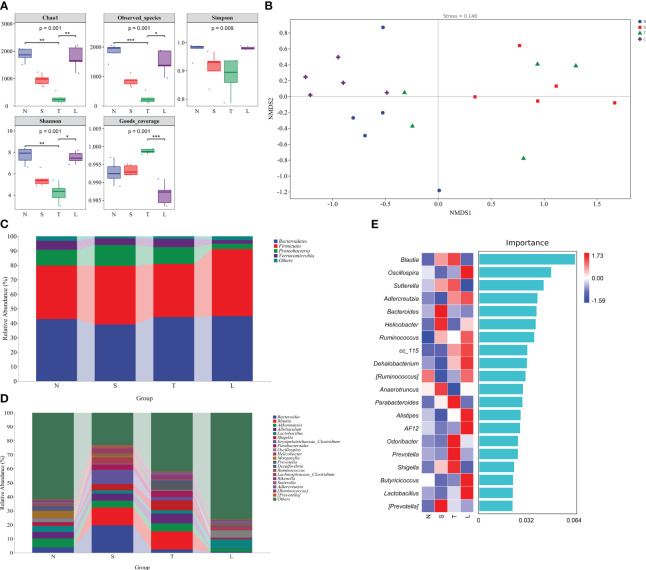
Oral administration of lysozyme alleviates gut dysbiosis. **(A)** Analysis of alpha diversity of gut microbiota. * *P* < 0.05; ** *P* < 0.01; *** *P* < 0.001. The data are presented as the means ± SD. **(B)** Analysis of beta diversity by NMDS. **(C)** Relative abundance of gut microbiota at phylum level. **(D)** Relative abundance of gut microbiota at genus level. **(E)** Random forest analysis. N, Normal group; S, Sham group; T, Severe traumatic brain injury group; L, Lysozyme treated group. NMDS, Nonmetric multidimensional scaling.

The alpha diversity of gut microbiota was analyzed in this study. Results demonstrated a significant reduction in the Chao1 index following sTBI (*P* < 0.05), which subsequently returned to normal levels after lysozyme treatment (**Figure 1A**). Similar trends were observed for the Observed species and Shannon index (**Figure 1A**). The Simpson index, although decreased, did not reach statistical significance (*P* > 0.05) (**Figure 1A**). Good’s coverage index showed an increase in sTBI mice and a subsequent decrease after lysozyme treatment, however, there was no statistically significant difference between N and T group (*P* > 0.05) (**Figure 1A**).

The beta diversity of gut microbiota was analyzed using NMDS. The NMDS analysis revealed significant differences between the T group and N group (Stress=0.148, R=0.36, P=0.022, Anosim), as well as between the N group and S group (R=0.72, P=0.009, Anosim) and L group (R=0.476, P=0.008, Anosim). The data showed in **Figure 1B**. The findings suggest that sTBI induces gut dysbiosis, and oral administration of lysozyme mitigates dysbiosis.

We further conducted an in-depth analysis of the classification of gut microbiota. The *Firmicutes*/*Bacteroidetes* ratio in the N group was 0.86, while it increased to 1.03 in the L group (**Figure 1C**). Moreover, there was a significant decrease in the relative abundance of *Proteobacteria* in group L, which decreased to 3.82% (**Figure 1C**). At the genus level, we observed a substantial increase in the relative abundance of *Blautia* in sTBI mice, reaching 12.79%, whereas it was almost undetectable in the L group. Additionally, *Allobaculum* showed a significant increase to 7.03% among sTBI mice compared to its lower presence in L group. *Shigella* was nearly absent from the L group but exhibited an increased relative abundance of 6.98% among sTBI mice compared to only 1% found within N group. Furthermore, *Parabacteroides* displayed an increased relative abundance of 4.38% among sTBI mice and a decreased presence down to 1.07% within the L group. Similarly, *Prevotella* showed an elevated relative abundance reaching up to 4.94% among sTBI mice and declined significantly down to 0.23% within L groups. The relative abundance of *Lactobacillus* in the N group was 4.31%, which decreased to 2.37% in sTBI mice, and increased to 6.44% following lysozyme treatment. The relative abundance of *Oscillospira* in the N group was 2.76%, which decreased to 0.72% in sTBI mice, while the relative abundance increased to 5.23% after treated with lysozyme. The data showed in **Figure 1D**.

The random forest analysis, a classical and efficient machine learning technique based on decision trees, is classified as a non-linear classifier capable of effectively capturing the intricate nonlinear relationships among variables. This characteristic makes it particularly suitable for analyzing microbial community data characterized by discrete and discontinuous distributions. Utilizing random forest analysis, we further validated that *Blautia*, *Sutterella*, *Parabacteroides*, *Prevotella*, and *Shigella* were the predominant bacteria in the gut microbiota of sTBI mice. Additionally, *Oscillospira*, *Adlercreutzia*, *Ruminococcus*, *Dehalobacterium*, *Alistipes*, *Butyricicoccus*, and *Lactobacillus* were identified as the major bacteria in mice treated with lysozyme. The data showed in **Figure 1E**.

### 3.2 Oral administration of lysozyme alleviates lung dysbiosis

**Figure 2:**
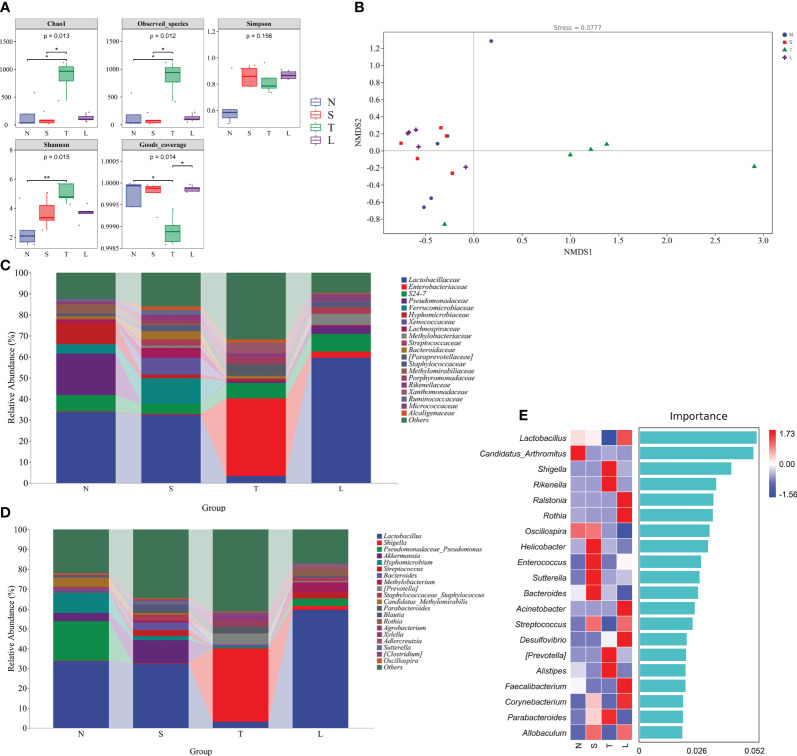
Oral administration of lysozyme alleviates lung dysbiosis. **(A)** Analysis of alpha diversity of lung microbiota. * *P* < 0.05; ** *P* < 0.01. The data are presented as the means ± SD. **(B)** Analysis of beta diversity by NMDS. **(C)** Relative abundance of lung microbiota at phylum. **(D)** Relative abundance of lung microbiota at genus. **(E)** Random forest analysis. N, Normal group; S, Sham group; T, Severe traumatic brain injury group; L, Lysozyme treated group. NMDS, Nonmetric multidimensional scaling.

The Chao1 index of lung microbiota exhibited a significant increase after sTBI (*P* < 0.05) and subsequently returned to baseline levels after administration of lysozyme (**Figure 2A**). The observed species index and Chao1 index exhibited a comparable pattern. The Shannon index of lung microbiota was elevated in sTBI mice but decreased in lysozyme treated mice (**Figure 2A**), whereas the Simpson index showed an increase without reaching statistical significance (*P* > 0.05) (**Figure 2A**). The Good’s coverage index significantly decreased in the lung microbiota of sTBI mice (*P* < 0.05), but was restored to normal levels after administration of lysozyme. These findings suggest that sTBI induces an increase in bacterial abundance and diversity in the mouse lungs, while concurrently reducing their coverage, indicating a potential overgrowth of specific bacterial species. The NMDS analysis revealed a significant distinction between the T group and the N group (Stress = 0.0777, R = 0.388, P=0.017, Anosim), while no significant differences were observed between the N group and either the S or L groups (*P* > 0.05, Anosim). These findings suggest that administration of lysozyme effectively mitigates lung dysbacteriosis. The data showed in **Figure 2B**.

The *Enterobacteriaceae* exhibited a relative abundance of up to 36.99% in the lungs of sTBI mice (**Figure 2C**). At the genus level, there was a significant increase in the relative abundance of pathogenic bacteria, including *Shigella* (36.88%), *Prevotella* (5.43%), and *Parabacteroides* (3.55%) in the lung of sTBI mice. In contrast, lysozyme-treated mice showed an elevated presence of beneficial bacteria such as *Lactobacillus* (59.41%), *Rothia* (3.89%), and *Adlercreutzia* (2.09%) in the lung. The data showed in **Figure 2D**. The results of the random forest analysis further confirmed that *Shigella*, *Prevotella*, *Alistipes*, and *Parabacteroides* were identified as the predominant bacterial species in the lungs of sTBI mice. Conversely, *Lactobacillus* and *Rothia* were found to be the dominant bacteria in the lungs of lysozyme-treated mice (**Figure 2E**).

### 3.3 Lysozyme restored intestinal barrier integrity and protected against lung injury and bacterial translocation

**Figure 3:**
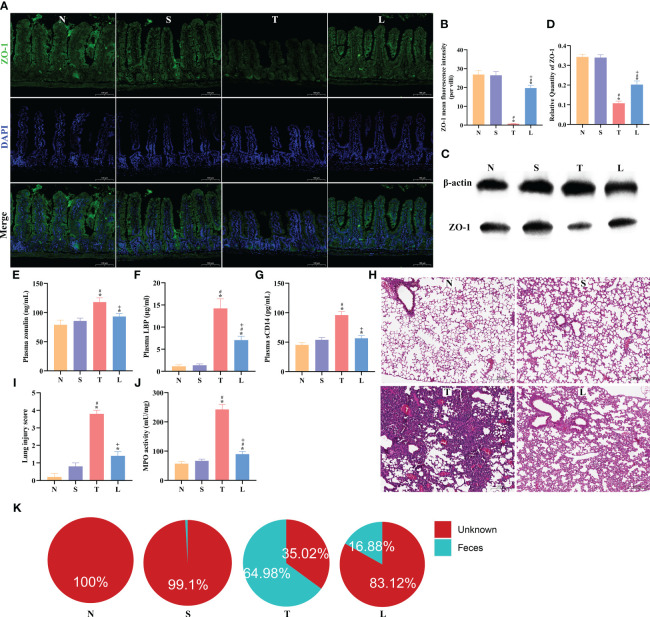
Lysozyme restored intestinal barrier integrity and protected against lung injury and bacterial translocation. **(A)** The immunofluorescence analysis of ZO-1 in ileum, scale bar: 100μm. **(B)** The mean fluorescence intensity per villi for ZO-1. **(C)** Western blot of ZO-1. **(D)** Quantification of Western blotting results. **(E)** Concentration of plasma Zonulin. **(F)** Levels of plasma LBP. **(G)** Levels of plasma sCD14. **(H)** Lung tissue HE staining, scale bar: 100 μm. **(I)** Quantification of lung injury score. **(J)** MPO activity of lung tissue. **(K)** SourceTracker identified lung microbiota contains bacteria from the gut microbiota. The data are presented as the means ± SD. ^*^ *P* < 0.05 compared to N, ^#^ *P* < 0.05 compared to S, ^+^ *P* < 0.05 compared to T. N, Normal group; S, Sham group; T, Severe traumatic brain injury group; L, Lysozyme treated group. LBP, lipopolysaccharide binding protein. MPO, Myeloperoxidase.

The protein ZO-1 is a crucial component of tight junctions, playing an essential role in the maintenance of cellular polarity and the integrity of the intestinal epithelial barrier. Compared with normal mice, the expression of ZO-1 in sTBI mice decreased significantly (*P* < 0.05), while the ZO-1 was increased after lysozyme administration (*P* < 0.05) (**Figures 3A–D**). There were no significant changes in ZO-1 expression levels in group NL and SL compared with group N or S, as detailed in **Supplementary Figures 1A–D**. The intestinal injury markers and bacterial translocation markers in the blood of mice were further examined. The levels of plasma Zonulin, LBP, and sCD14 were significantly increased in mice with sTBI compared to those in group N or group S (*P* < 0.05) (**Figures 3E–G**). Compared with sTBI mice, the levels of Plasma Zonulin, LBP, and sCD14 exhibited a significant decrease (*P* < 0.05) (**Figures 3E–G**). Plasma Zonulin, LBP, and sCD14 expression levels were not significantly changed in group NL and SL compared with group N or S, as detailed in **Supplementary Figures 1E–G**.

After sTBI, lung histopathological examination showed alveolar collapse and extensive infiltration of inflammatory cells. The injury was alleviated after administration of lysozyme (**Figure 3H**). The pathological evaluation indicated a significant increase in the lung injury score post-sTBI (*P* < 0.05), which significantly decreased after administration of lysozyme compared to the T group (*P* < 0.05) (**Figure 3I**). Nevertheless, it did not completely return to the levels in the N or S group (**Figure 3I**). Further analysis of MPO levels in lung tissues revealed a significant elevation in the T group compared to the N and S group (*P* < 0.05), while the MPO level was reduced compared to the T group following administration of lysozyme (**Figure 3J**). Lung injury score and MPO levels were not significantly changed in group NL and SL compared with group N or S, as detailed in **Supplementary Figures 1H–J**. These findings suggest that administration of lysozyme can mitigate lung injury severity after sTBI. SourceTracker analysis predicted a gut origin for lung bacteria, with 64.98% originating from the gut after sTBI (**Figure 3K**), which decreased to 16.88% after administration of lysozyme (**Figure 3K**).

### 3.4 Lysozyme ameliorated sTBI-induced ileum inflammation and Paneth cells dysfunction

**Figure 4:**
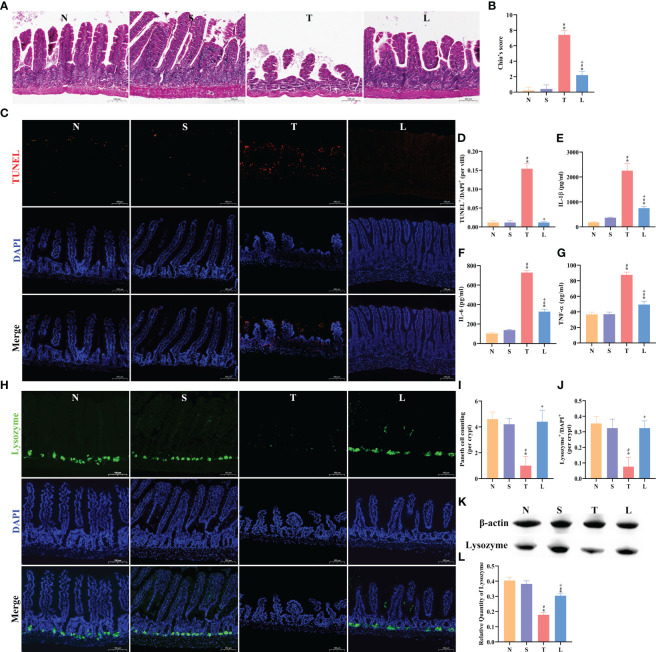
Lysozyme ameliorated sTBI-induced ileum inflammation and Paneth cell dysfunction. **(A)** The HE staining of ileum tissue, scale bar: 100 μm. **(B)** Chiu’s score of ileum tissue. **(C)** The TUNEL staining of ileum tissue, scale bar: 100 μm. **(D)** TUNEL^+^/DAPI^+^ quantification of apoptosis in the ileum per villi. **(E–G)** Concentration of IL-1β, IL-6 and TNF-α in ileum. **(H)** The immunofluorescence staining of lysozyme, scale bar: 100 μm. **(I)** Mean number of Paneth cells per crypt. **(J)** Lysozyme^+^/DAPI^+^ quantification. **(K)** Western blotting of Lysozyme. **(L)** Quantification of Western blotting results. The data are presented as the means ± SD. ^*^ *P* < 0.05 compared to N, ^#^ *P* < 0.05 compared to S, ^+^ *P* < 0.05 compared to T. N, Normal group; S, Sham group; T, Severe traumatic brain injury group; L, Lysozyme treated group.

The HE staining of the ileum revealed muscular layer atrophy and intestinal villus damage following sTBI (**Figure 4A**). Chiu’s score demonstrated a significantly higher score in the T group compared to the N or S group (*P* < 0.05). Administration of lysozyme effectively mitigated the severity of ileal injury (*P* < 0.05) (**Figure 4B**). Furthermore, we assessed apoptosis levels in the ileum, which showed a significant increase after sTBI compared to the N or S group (*P* < 0.05) (**Figures 4C, D**). Treatment with lysozyme reduced apoptosis levels (**Figures 4C, D**). Oral administration of lysozyme was not significantly affected in the ileum of normal or sham mice, as detailed in **Supplementary Figures 2A–D**. We further examined the levels of inflammatory factors in the ileum tissue. Compared with normal mice, IL-1β, IL-6 and TNF-α in ileum of sTBI mice were significantly increased (*P* < 0.05) (**Figures 4E–G**). Administration of lysozyme significantly reduced the level of intestinal inflammation (*P* < 0.05) (**Figures 4E–G**). Oral administration of lysozyme had no significant effect on the expression of IL-1β, IL-6 and TNF-α in the ileum of normal or sham mice, as detailed in **Supplementary Figures 2E–G**. The function of Paneth cells plays an important role in the regulation of gut microbiota and intestinal barrier. Compared with normal mice, the number of Paneth cells in sTBI mice decreased significantly (*P* < 0.05) and the level of lysozyme in sTBI mice decreased significantly (*P* < 0.05) (**Figures 4H–L**). The number of Paneth cells and the expression of lysozyme in mice treated with lysozyme returned to the normal level (*P* < 0.05) (**Figures 4H–L**). Oral administration of lysozyme had no significant effect on Paneth cells in the ileum of normal or sham mice, as detailed in **Supplementary Figures 2H–L**.

### 3.5 The recovery of Paneth cells function by oral administration of lysozyme depends on gut microbiota

**Figure 5:**
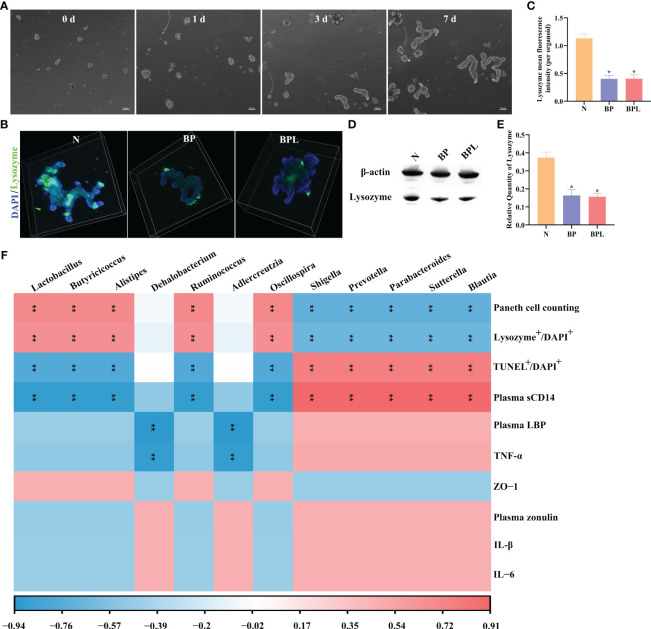
Intestinal organoids co-culture system and Correlation analysis. **(A)** Light microscopy photographs showing normal intestinal organoid, scale bar: 100 μm. **(B)** The immunofluorescence staining of lysozyme in organoids. **(C)** The mean fluorescence intensity of lysozyme per organoid. **(D, E)** Western blotting and relative quantity of lysozyme. The data are presented as the means ± SD. ^*^ *P* < 0.05 compared to N. **(F)** The correlation between ileal pathological indicators and gut microbiota by Spearman correlation coefficient. Red indicated a positive correlation. Blue indicated a negative correlation. Color depth indicates the strength of correlation. **P < 0.01. N, Normal group; BP, Brain-derived proteins + Intestinal organoids co-culture; BPL, Brain-derived proteins + Lysozyme + Intestinal organoids co-culture. BP, Brain-derived proteins.

We established an intestinal organoids culture system and cultured for 0-7 days (**Figure 5A**). Co-culturing brain-derived proteins (BP) with intestinal organoids resulted in a significant reduction of lysozyme expression (*P* < 0.05) (**Figures 5B, C**). However, adding lysozyme to the BP co-cultured intestinal organoids did not increase the expression level of lysozyme in organoids (**Figures 5B, C**). The expression level of lysozyme in organoids was further assessed using western blotting analysis. The results demonstrated a significant reduction in lysozyme expression after treatment with BP (*P* < 0.05), while the addition of exogenous lysozyme did not result in an increase in lysozyme expression within organoids (*P* > 0.05) (**Figures 5D, E**). Adding lysozyme to the normal cultured intestinal organoids did not increase the expression level of lysozyme in organoids, as detailed in **Supplementary Figures 3A–E**. The findings suggest that the exogenous lysozyme does not effectively restore the functionality of damaged Paneth cells.

Correlation analysis was conducted to examine the relationship between gut microbiota identified by random forest analysis and ileal pathological indicators. In sTBI mice, the gut microbiota (*Blautia*, *Sutterella*, *Parabacteroides*, *Prevotella*, and *Shigella*) exhibited a negative correlation with the number of Paneth cells (*P* < 0.01) or lysozyme expression (*P* < 0.01), while showing a positive correlation with apoptosis of ileal cells (*P* < 0.01) and the level of plasma sCD14 (*P* < 0.01). In lysozyme treated mice, there was a positive correlation between gut microbiota (*Oscillospira*, *Ruminococcus*, *Alistipes*, *Butyricicoccus*, and *Lactobacillus*) and the number of Paneth cells (*P* < 0.01) or the expression level of lysozyme (*P* < 0.01). Conversely, these microbes were negatively correlated with ileal cell apoptosis or plasma sCD14 (*P* < 0.01). *Adlercreutzia* and *Dehalobacterium* exhibited negative correlations with plasma LBP and TNF-α (*P* < 0.01). The data showed in **Figure 5F**. The aforementioned findings demonstrate that the modulation of gut microbiota (including *Oscillospira*, *Ruminococcus*, *Alistipes*, *Butyricicoccus*, and *Lactobacillus*) in lysozyme-treated mice may exert a pivotal role in regulating and ameliorating intestinal barrier damage, as well as modulating Paneth cells.

## 4 Discussion

The present study provides evidence that lysozyme possesses the ability to rectify the dysbiosis of gut microbiota induced by sTBI, restore lung microbiota composition, and mitigate translocation of intestinal bacteria. Oral administration of lysozyme reinstates lysozyme expression in Paneth cells, thereby reducing intestinal permeability, pathological score, apoptosis rate, and inflammation levels. We established a co-culture system comprising intestinal organoids and BP, which demonstrated that the BP effectively downregulated the expression of lysozyme in intestinal organoids. However, supplementation of lysozyme to this co-culture system failed to restore its expression in intestinal organoids. We conducted a more in-depth analysis to explore the correlation between Paneth cells and lysozyme expression levels, as well as their relationship with gut microbiota. There was a positive correlation between gut microbiota (*Oscillospira*, *Ruminococcus*, *Alistipes*, *Butyricicoccus*, and *Lactobacillus*) and the number of Paneth cells or the expression level of lysozyme. Conversely, these microbes were negatively correlated with ileal cell apoptosis or plasma sCD14. The present study unveiled a virtuous cycle whereby lysozyme restores Paneth cell’s function, mitigates intestinal injury and bacterial translocation through the remodeling of gut microbiota.

sTBI is a major cause of death and disability worldwide (Stocchetti et al., 2017). Acute brain injury, such as sTBI, is complicated by neuroinflammation and neurodegeneration, leading to multiorgan inflammation, microbial dysbiosis, gastrointestinal dysfunction and dysmotility, hepatic dysfunction, acute renal injury, and cardiac dysfunction (Krishnamoorthy et al., 2021; Battaglini et al., 2023). The resultant dysautonomia and systemic inflammation caused by TBI contribute to secondary gastrointestinal events, such as dysmotility and increased mucosal permeability (Hanscom et al., 2021). Our previous study showed intestinal barrier damage and reduced antimicrobial peptide secretion in TBI mice (Yang et al., 2022). Animal models of TBI have been well established and widely recognized. Intestinal injury caused by TBI has been extensively studied. The study conducted by You et al. demonstrated that TBI induces intestinal inflammation and compromises the integrity of the gut barrier in mice (You et al., 2022). Mazarati A et al. demonstrated that TBI induces early and distant disruption of the intestinal barrier, leading to a subsequent endotoxemia (Mazarati et al., 2021). As Ma EL et al. demonstrated, TBI altered colon morphology by thickening smooth muscles and increasing mucosal depth (Ma et al., 2017). On day 28 post-TBI, there was a decrease in the expression of claudin-1 mRNA and protein and an increase in paracellular permeability. Clinical or animal studies of TBI induced lung injury have been widely reported. The main pulmonary disorders that occur after TBI includes neurogenic pulmonary edema, acute respiratory distress syndrome, and ventilator-associated pneumonia. Similarly, the primary brain disorders that arise after lung injuries encompass brain hypoxia and intracranial hypertension (Chacon-Aponte et al., 2022). It was reported that TBI resulted in pulmonary injury in mice (Kerr et al., 2018; Kerr et al., 2021).

Considering the well-established antibacterial, antiviral, antifungal, anti-inflammatory, anticancer, and immunomodulatory properties, lysozyme exhibits significant potential in clinical settings as well as in feed and food applications for combating diverse pathogens (Ferraboschi et al., 2021). The production of lysozyme, a key component of innate immunity, is primarily attributed to Paneth cells located in the small intestine (Bevins and Salzman, 2011; Brott and Clarke, 2019). Yang F et al. demonstrated that administration of lysozyme effectively ameliorated intestinal inflammation through the restoration of Paneth cell functionality (Fu et al., 2022). Larsen S et al. demonstrated that oral administration of lysozyme effectively mitigated intestinal inflammation in mice (Larsen et al., 2021). Our study demonstrated that sTBI-induced ileal injury was accompanied by an inflammatory response, and oral administration of lysozyme restored the expression of lysozyme in Paneth cells, thereby alleviating both ileal injury and inflammation. Yu SY et al. demonstrated that lysozyme exerts regulatory effects on intestinal mucosal inflammation and immunity through modulation of the gut microbiota (Yu et al., 2020). The mechanisms underlying the ability of lysozyme to limit intestinal inflammation remain unclear. Ragland SA, et *al.* conducted a comprehensive review to explore various potential mechanisms underlying the anti-inflammatory effects of lysozyme (Ragland and Criss, 2017).

Paneth cells play a pivotal role in maintaining intestinal homeostasis, and their dysfunction is implicated not only in intestinal disorders but also in extraintestinal pathologies (Cui et al., 2023). Paneth cells exert regulatory control over the abundance of mucosa-associated bacteria, thereby preventing direct contact with the epithelium and subsequent translocation of bacterial species (Wallaeys et al., 2023). Additionally, Paneth cells are non-proliferative intestinal epithelial cells that provide support for the stem cell microenvironment (Yu et al., 2018). The Paneth cells, which express EGF, TGF-α, Wnt3, and the Notch ligand Dll4, constitute the niche for Lgr5 stem cells in intestinal crypts (Sato et al., 2011a). Therefore, Paneth cells play a crucial role in regulating the proliferation of stem cells and maintaining the integrity of the intestinal barrier (Barreto et al., 2022). Supplementation of Paneth cell-derived products is a widely employed approach in research pertaining to the dysfunction of these cells, aiming at promoting functional recovery (Schoenborn et al., 2019; Jiang et al., 2021). We found that lysozyme treatment was able to restore the function of Paneth cells in mice is similar to the findings of others (Fu et al., 2022). In addition, our study demonstrated that administration of lysozyme not only restores Paneth cells’ function but also mitigates small intestinal injury.

Epithelial organoids are already demonstrating their transformative impact in basic and translational research (Gjorevski et al., 2022). Our study demonstrated that lysozyme administration restored the number of Paneth cells and lysozyme expression in the small intestine of sTBI mice. However, it remains unclear whether these findings are attributed to the direct impact of exogenous lysozyme or alterations in gut microbiota. Therefore, intestinal organoids were used in our study. The cellular composition of small intestinal organoids consists of mature enterocytes, goblet cells, enteroendocrine cells, and Paneth cells (Sato et al., 2011b). We demonstrated that co-culturing BP with intestinal organoids resulted in a significant reduction in the expression of lysozyme within the organoids, indicating impaired Paneth cell’s function. As it is widely acknowledged, brain injury induces alterations in peripheral serotonin homeostasis, thereby potentially influencing gastrointestinal function, gut microbiota composition, and systemic energy balance (Mercado et al., 2022). The sympathetic and parasympathetic nervous systems play a crucial role in modulating various gut functions, including regional motility, intestinal permeability, immune response of the mucosa, maintenance of epithelial fluid balance, as well as the production of gastric acid, mucus, bicarbonate, gut peptides and antimicrobial peptides (Dabrowski et al., 2021). Our co-culture of BP with intestinal organoids resulted in a downregulation of lysozyme expression. The findings suggest that BP has the potential to induce dysfunction in Paneth cells, irrespective of the involvement of the neuroendocrine system, thereby potentially contributing to intestinal injury after TBI. Further investigation is warranted to elucidate this underlying mechanism. The supplementing of lysozyme to the aforementioned culture system did not yield any improvement in the expression of lysozyme in organoids. In conjunction with *in vivo* experiments, it was demonstrated that the restoration of Paneth cell’s function by lysozyme supplementation was dependent on the presence of gut microbiota. The mechanism of lysozyme secretion by Paneth cells has not been fully elucidated, but current understanding suggests that the regulation of lysozyme secretion is primarily influenced by bacteria and their metabolites (Zhang and Liu, 2016). Our study further substantiates this concept; however, additional investigation is required to elucidate the regulatory mechanisms of gut microbiota on Paneth cell functionality.

The gut microbiota plays a crucial role in maintaining the physiological structure and function of the intestines, which is closely associated with intestinal inflammation, barrier integrity, and host growth performance (Adak and Khan, 2019). The alpha diversity refers to the diversity in a specific region or ecosystem, which is often measured by Chao 1, Observed species, Simpson, Shannon indicators and Good’s coverage (Nowicki et al., 2023). Among them, the Chao1 index and Observed species are related to microbial community richness, the Shannon index and Simpson index are related to the diversity of intestinal flora, Good’s coverage characterizes the degree of coverage. We have demonstrated that the administration of lysozyme contributes to the restoration of gut microbial diversity, aligning with previous findings by others (Fu et al., 2022).

The *Firmicutes/Bacteroidetes* ratio is widely accepted to have an important influence in maintaining normal intestinal homeostasis (Stojanov et al., 2020). We found that administration lysozyme increased the relative abundance of *Firmicutes* and the ratio of *Firmicutes/Bacteroidetes*. The results are consistent with those reported in the literatures (Kim et al., 2023; Wu et al., 2023). *Firmicutes* has a variety of effects on the body. Lysozyme facilitates the translocation of peptidoglycan from *Firmicutes* across the intestinal epithelium, promoting integration of *Firmicute* cell wall glycoconjugates into systemic immunity (Jordan et al., 2023). This integration enhances defenses against pneumonia, sepsis, and meningitis. The *Firmicutes* have been shown to significantly enhance the local production of butyrate, which is widely acknowledged for its robust association with a healthy immune homeostasis and safeguarding against inflammatory disorders (Leth et al., 2023). *Proteobacteria* are frequently overrepresented in various intestinal and extraintestinal diseases, predominantly exhibiting an inflammatory phenotype (Rizzatti et al., 2017). The results of our study demonstrated that administration of lysozyme significantly decreased the relative abundance of *Proteobacteria*.

At the genus level, both random forest analysis and correlation analysis confirmed that *Lactobacillus*, *Butyricicoccus*, *Alistipes*, *Ruminococcus*, and *Oscillospira* exhibited positive correlations with the number of Paneth cells and the expression of lysozyme while showing negative correlations with TUNEL or plasma sCD14. However, the addition of exogenous lysozyme did not restore the functionality of Paneth cells in the organoids co-culture system. These findings suggest that administration of lysozyme can restore Paneth cells function by regulating the composition of gut microbiota. Therefore, the aforementioned bacteria may play a crucial role in modulating Paneth cell functionality and ameliorating damage to the intestinal barrier.

*Lactobacillus* generates lactic acid in the intestinal lumen, which binds to lactate G-protein-coupled receptor and subsequently stimulates Paneth cells to upregulate wingless-related integration site 3a expression (Lee et al., 2018). This leads to the regulation of self-renewal, differentiation, and crypt formation of Lgr5+ intestinal stem cells (Lee et al., 2018). Additionally, the pili of *Lactobacillus* have demonstrated anti-inflammatory effects by suppressing the production of proinflammatory cytokines and promoting the secretion of anti-inflammatory cytokines such as IL-10, thereby mitigating excessive inflammation and maintaining tissue homeostasis (Lee et al., 2023). Clinical studies have demonstrated that *Butyricicoccus*, which is responsible for butyrate production, exhibits increased abundance in patients with Crohn’s disease following treatment (Facchin et al., 2020). This suggests that *Butyricicoccus* exerts multiple beneficial effects including the amelioration of inflammation, enhancement of oxidative status, reinforcement of epithelial defense barrier, and modulation of visceral sensitivity and intestinal motility (Facchin et al., 2020). Additionally, the presence of *Butyricicoccus*, a bacterial genus associated with the mucosa, further supports its potential as a pharmabiotic for preserving the integrity of epithelial tight junctions (Devriese et al., 2017). It was reported that *Alistipes* dysbiosis can be either beneficial, or harmful(Parker et al., 2020). The evidence also demonstrates that *Alistipes* functions as a producer of short chain fatty acids, thereby suggesting its potential involvement in anti-inflammatory mechanisms (Li et al., 2016; Oliphant and Allen-Vercoe, 2019). *Ruminococcus* are now acknowledged as significant contributors to the gut ecosystem and have acquired the capacity to break down and utilize a wide range of plant polysaccharides, which holds implications for both host health and potential biotechnological exploitation (La Reau and Suen, 2018). *Oscillospira* frequently constitutes a substantial proportion of the fecal microbiome in humans, occasionally surpassing 10% of individual 16S rRNA gene sequences (Konikoff and Gophna, 2016). *Oscillospira* is likely to be a producer of the short-chain fatty acid butyrate and holds promise as a potential candidate for next-generation probiotics (Yang et al., 2021). The results of our study indicate that *Alistipes*, *Ruminococcus*, and *Oscillospira* are distinct bacterial species present in the gut microbiota of lysozyme-treated mice. Furthermore, these bacteria demonstrate a positive association with Paneth cell functionality and a negative association with intestinal apoptosis.

Conversely, *Blautia*, *Sutterella*, *Parabacteroides*, *Prevotella*, and *Shigella* displayed negative correlations with the number of Paneth cells or lysozyme expression but showed positive correlations with TUNEL or plasma sCD14 expression. The CD14 molecule is a glycosyl phosphatidylinositol surface-anchored protein that is predominantly expressed by myeloid cells, specifically monocytes/macrophages and to a lesser extent, neutrophils (Leveque et al., 2017). The cell types, such as hepatocytes and enterocytes, have the ability to directly produce sCD14 and secrete it into the bloodstream independently of its presence on the cell surface (Na et al., 2023). The increased levels of sCD14 have been reported to be associated with the activation of myeloid cells by lipopolysaccharide (LPS), which is the principal constituent of the outer membrane in Gram-negative bacteria (Zanoni and Granucci, 2013). However, *Streptococcus*, *Shuttleworthia*, and *Rothia* are classified as Gram-positive bacteria instead of Gram-negative bacteria and have been reported to be associated with increased plasma sCD14 levels in recent literature (Maccioni et al., 2020). Furthermore, levels of sCD14 exhibit an elevation in both acute and chronic inflammatory conditions and have been associated with diseases and risk factors characterized by inflammation (Stanislawski et al., 2021). The activation of sCD14 by factors derived from both pathogens and tissue damage can initiate signaling in a diverse range of non-immune cells (Sharygin et al., 2023). The plasma sCD14 concentrations are associated with various factors, including LPS, peptidoglycan, lipoteichoic acid, and tissue damage (Dessing et al., 2007; Wu et al., 2019). The findings of our research indicate a positive correlation between the level of plasma sCD14 and Blautia, a gram-positive bacterium. *Blautia* exhibits the capacity to produce short-chain fatty acids; however, the proportion of genes associated with carbohydrate transport and metabolism in *Blautia* is relatively small (6.57%), suggesting a limited ability of *Blautia* to metabolize carbohydrates (de Mooij et al., 2023). Additionally, there exists a disparity in the association of the *Blautia* genus with human diseases. Notably, *Blautia* exhibits a positive correlation with key inflammatory cytokines such as TNF-α (Barber et al., 2023). Moreover, *Blautia* may potentially contribute to the bioconversion of primary bile acids into secondary bile acids, which have been documented as deleterious and even carcinogenic substances (Gomez-Perez et al., 2023). The findings of our study demonstrated a significant negative association between *Blautia* and both the quantity and functionality of Paneth cells. Moreover, *Blautia* exhibited a weak positive correlation with TNF-α levels. To date, only a limited number of strains of *Blautia*, which is strictly anaerobic bacteria, have been isolated. Furthermore, Bergey’s manual does not provide any description of the characteristics of this genus and its genomic information remains scarce (Liu et al., 2021). The correlation between *Blautia* and host health requires further research in order to achieve a comprehensive understanding.

The presence of elevated levels of *Sutterella* in the gut microbiome exhibited an inverse correlation with fecal IgA levels, suggesting a potential role for *Sutterella* in the degradation of IgA (Moon et al., 2015). When co-cultured with intestinal epithelial cells, *Sutterella* exhibited a proinflammatory capacity without compromising the integrity of the monolayer (Kaakoush, 2020). Our study revealed a significant inverse association between *Sutterella* and the number and functionality of Paneth cells. Although there was a negative correlation between *Sutterella* and ZO-1, no statistically significant difference was observed. *Parabacteroides* primarily act as opportunistic pathogens and exhibit a strong association with chronic inflammatory conditions, as well as heightened abundance in patients suffering from brain injuries. Additionally, it is imperative to acknowledge the antimicrobial resistance of *Parabacteroides* towards antibiotics such as clindamycin, moxifloxacin, and cefoxitin (Cui et al., 2022). *Prevotella* has been implicated in a multitude of diseases, encompassing inflammatory autoimmune disorders, opportunistic infections, as well as oral biofilm formation and associated pathologies (Tett et al., 2021). *Prevotella* predominantly activate Toll-like receptor 2, leading to production of IL-23 and IL-1 (Larsen, 2017). Moreover, *Prevotella* modulates the composition and functionality of the gut microbiota, leading to a decline in levels of short-chain fatty acids, particularly acetate (Iljazovic et al., 2021). Previous studies have demonstrated a substantial increase in resistance of *Prevotella* to several antibiotics (clindamycin, penicillin, cephalosporin, tetracycline, amoxicillin, and metronidazole), attributed to the presence of antimicrobial resistance genes or efflux pumps (Sharma et al., 2022). The *Shigella* bacteria reside in the gastrointestinal tract and are the primary causative agents of bacillary diarrhea or shigellosis in humans (Pakbin et al., 2023). The involvement of increased *Shigella* and decreased lysozyme in the pathogenesis of intestinal barrier dysfunction during acute necrotizing pancreatitis is evident (Chen et al., 2017). The adult mice’s intestinal tract exhibits inherent resistance to *Shigella* infection (Fernandez et al., 2008). Conversely, neonatal mice exhibit a heightened susceptibility to intragastric *Shigella* infection, leading to the development of inflammatory lesions in the jejunal mucosa (Fernandez et al., 2008). The pivotal role of Paneth cell maturation becomes indispensable for effective clearance of intestinal infections (Fernandez et al., 2008).

Dysbiosis-induced proliferation of gut bacteria can elicit pulmonary inflammatory responses, thereby contributing to the pathogenesis of both acute and chronic respiratory disorders (Belizario et al., 2023). Although the gut and lung are anatomically distinct, the presence of potential anatomic communications and intricate pathways involving their respective microbiota has substantiated the existence of a gut-lung axis (Enaud et al., 2020). It was reported that early-life gut colonization can exert distal effects on the lung (Tamburini and Clemente, 2017). The resistance of neonatal mice to pneumonia is dependent on the recruitment of intestinal commensal bacteria-directed group 3 innate lymphoid cells into the lungs, which in turn mediate IL-22-dependent host defense against pneumonia (Gray et al., 2017). These data establish that postnatal colonization by intestinal commensal bacteria is pivotal in the development of the lung defenses of newborns. In addition, impairment of the gut barrier facilitates the translocation of microbial entities (living organisms or cellular fragments) to the pulmonary system (Nath et al., 2023). Interactions between the host and gut microbiota within the mucosal lining of the gastrointestinal tract can exert an influence on lung physiology via secretion of microbial metabolites or transmission of host-derived messengers such as hormones, cytokines, or immune cells (Ma et al., 2022). Persistent disruption of the intestinal barrier can result in translocation of microbial components into the systemic circulation, thereby inducing a state of chronic, low-grade inflammation (Martel et al., 2022). The dysbiosis of gut microbiota directly contributes to increased gut permeability, thereby facilitating the translocation of pathogenic bacteria and allowing the translocation of bacterial products from the gut into circulation (Chen et al., 2022). Zonulin, LBP and sCD14 have been identified as reliable indicators of gut permeability and bacterial translocation (Kavanagh et al., 2019; Palomino-Kobayashi et al., 2022). The Zonulin can be detected in plasma and exhibit a strong correlation with bacterial DNAemia, making it a valuable biomarker for assessing impaired gut barrier function (Veres-Szekely et al., 2023). LBP is mainly produced in hepatocytes and plays a pivotal in the innate immune response for the development of inflammatory and infectious-related diseases (Meng et al., 2021). We demonstrated that oral administration of lysozyme ameliorates the dysbiosis of gut microbiota induced by sTBI, restores the composition of lung microbiota, and mitigates the translocation of intestinal bacteria.

In conclusion, the present study unveiled a virtuous cycle whereby lysozyme restores Paneth cell’s function, mitigates intestinal injury and bacterial translocation through the remodeling of gut microbiota.

## Data availability statement

The datasets presented in this study can be found in online repositories. The names of the repository/repositories and accession number(s) can be found below: https://www.ncbi.nlm.nih.gov/, PRJNA1017641.

## Ethics statement

The animal study was approved by the ethics committee of Huashan Hospital Affiliated to Fudan University. The study was conducted in accordance with the local legislation and institutional requirements.

## Author contributions

WY: Data curation, Funding acquisition, Methodology, Writing – original draft. CX: Data curation, Methodology, Writing – review & editing. HY: Formal Analysis, Writing – review & editing. QY: Formal Analysis, Writing – review & editing. JZ: Formal Analysis, Visualization, Writing – review & editing. QC: Methodology, Writing – review & editing. GW: Conceptualization, Project administration, Supervision, Writing – review & editing. JH: Conceptualization, Funding acquisition, Project administration, Supervision, Writing – review & editing.

## Supplementary material

Supplementary Figure 1 Lysozyme had no effect on the small intestinal barrier and lung in normal mice. **(A)** The immunofluorescence analysis of ZO-1 in ileum, scale bar: 100 μm. **(B)** The mean fluorescence intensity per villi for ZO-1. **(C)** Western blotting of ZO-1. **(D)** Quantification of Western blotting results. **(E)** Concentration of plasma Zonulin. **(F)** Levels of plasma LBP. **(G)** Levels of plasma sCD14. **(H)** Lung tissue HE staining, scale bar: 100 μm. **(I)** Quantification of lung injury score. **(J)** MPO activity of lung tissue. The data are presented as the means ± SD. ^*^ *P* < 0.05 compared to N, ^#^ *P* < 0.05 compared to S, ^+^ *P* < 0.05 compared to T, ^&^ *P* < 0.05 compared to L. N, Normal group; S, Sham group; T, Severe traumatic brain injury group; L, Lysozyme treated group; NL: Normal + Lysozyme group; SL: Sham group + Lysozyme group. LBP, lipopolysaccharide binding protein. MPO, Myeloperoxidase.

Supplementary Figure 2 Lysozyme had no effect on normal ileum and Paneth cell’s function. **(A)** The HE staining of ileum tissue, scale bar: 100μm. **(B)** Chiu’s score of ileum tissue. **(C)** The TUNEL staining of ileum tissue, scale bar: 100μm. **(D)** TUNEL^+^/DAPI^+^ quantification of apoptosis in the ileum per villi. **(E-G)** Concentration of IL-1β, IL-6 and TNF-α in ileum. **(H)** The immunofluorescence staining of lysozyme, scale bar: 100μm. **(I)** Mean number of Paneth cells per crypt. **(J)** Lysozyme^+^/DAPI^+^ quantification. **(K)** Western blot of Lysozyme. **(L)** Quantification of western blot results. The data are presented as the means ± SD. ^*^ *P* < 0.05 compared to N, ^#^ *P* < 0.05 compared to S, ^+^ *P* < 0.05 compared to T, ^&^ *P* < 0.05 compared to L. N, Normal group; S, Sham group; T, Severe traumatic brain injury group; L, Lysozyme treated group; NL: Normal + Lysozyme group; SL: Sham group + Lysozyme group.

Supplementary Figure 3 Lysozyme had no effect on normal Intestinal organoids function. **(A)** Light microscopy photographs showing normal intestinal organoid, scale bar: 100μm. **(B)** The immunofluorescence staining of lysozyme in organoids. **(C)** The mean fluorescence intensity of lysozyme per organoid. **(D, E)** Western blotting and relative quantity of lysozyme. The data are presented as the means ± SD. ^*^ *P* < 0.05 compared to N, ^#^ *P* < 0.05 compared to BP, ^+^ *P* < 0.05 compared to BPL. N, Normal group; BP: Brain-derived proteins + Intestinal organoids co-culture; BPL, Brain-derived proteins + Lysozyme + Intestinal organoids co-culture. BP, Brain-derived proteins.
