# Supplementary figures and images for: Oral administration of lysozyme protects against injury of ileum via modulating gut microbiota dysbiosis after severe traumatic brain injury
Source: Front Cell Infect Microbiol. 2024 Jan 30;14:1304218. doi: 10.3389/fcimb.2024.1304218 (PMC10861676; doi:10.3389/fcimb.2024.1304218)

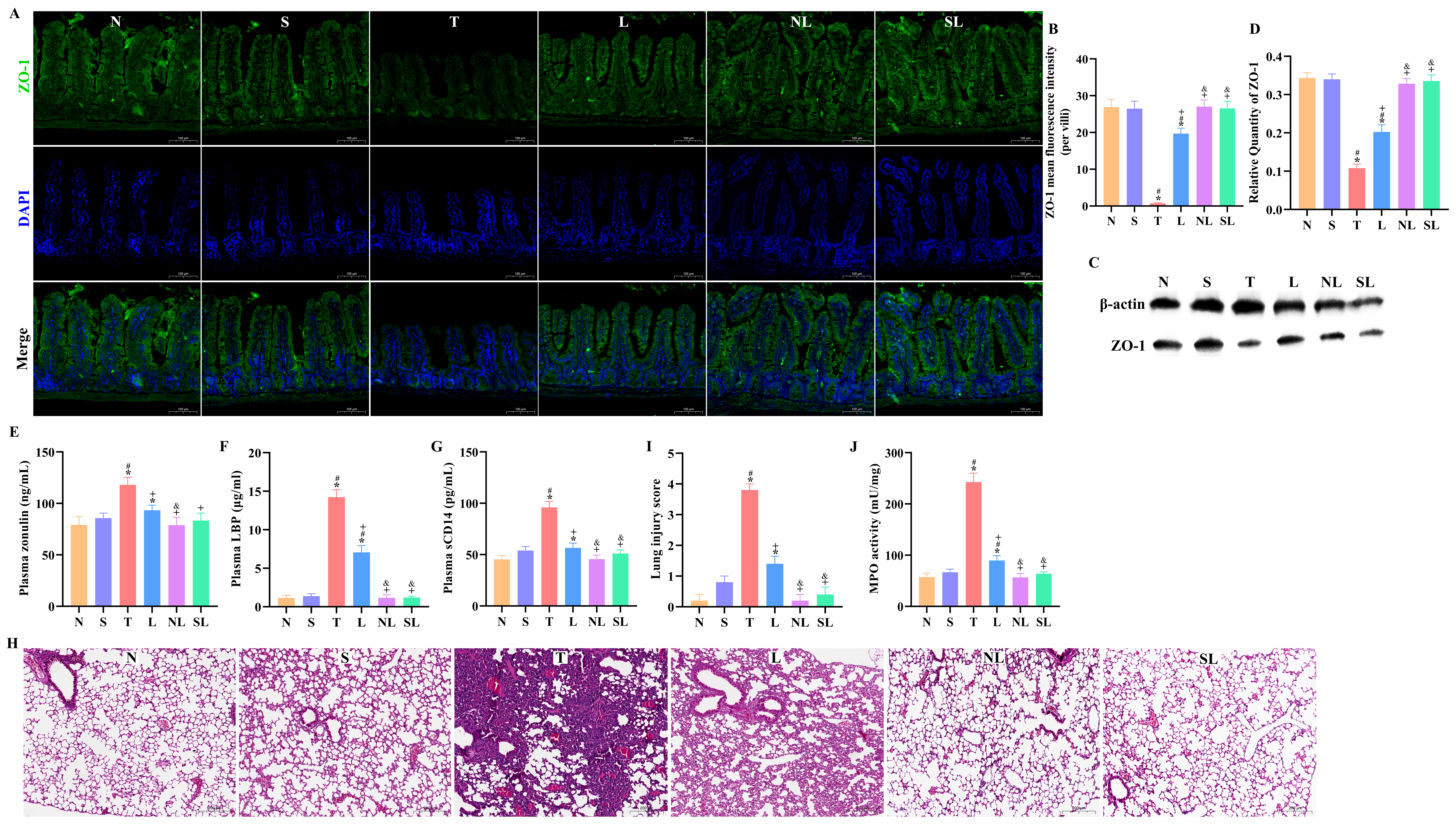

Supplement: Supplementary Figure 1 — Lysozyme had no effect on the small intestinal barrier and lung in normal mice. (A) The immunofluorescence analysis of ZO-1 in ileum, scale bar: 100 μm. (B) The mean fluorescence intensity per villi for ZO-1. (C) Western blotting of ZO-1. (D) Quantification of Western blotting results. (E) Concentration of plasma Zonulin. (F) Levels of plasma LBP. (G) Levels of plasma sCD14. (H) Lung tissue HE staining, scale bar: 100 μm. (I) Quantification of lung injury score. (J) MPO activity of lung tissue. The data are presented as the means ± SD. * P < 0.05 compared to N, # P < 0.05 compared to S, + P < 0.05 compared to T, & P < 0.05 compared to L. N, Normal group; S, Sham group; T, Severe traumatic brain injury group; L, Lysozyme treated group; NL: Normal + Lysozyme group; SL: Sham group + Lysozyme group. LBP, lipopolysaccharide binding protein. MPO, Myeloperoxidase. [file Image_1.tif]

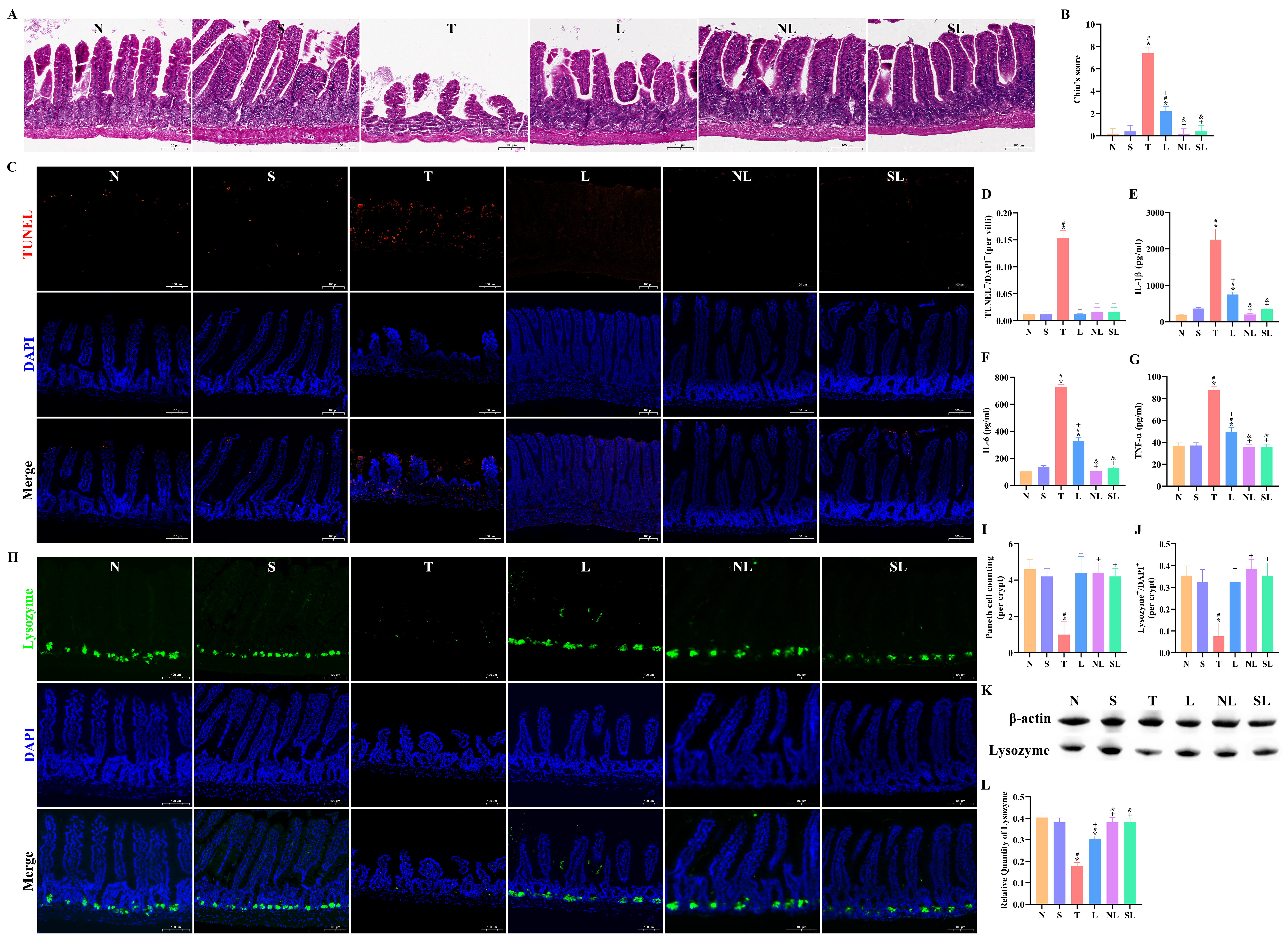

Supplement: Supplementary Figure 2 — Lysozyme had no effect on normal ileum and Paneth cell’s function. (A) The HE staining of ileum tissue, scale bar: 100μm. (B) Chiu’s score of ileum tissue. (C) The TUNEL staining of ileum tissue, scale bar: 100μm. (D) TUNEL+/DAPI+ quantification of apoptosis in the ileum per villi. (E-G) Concentration of IL-1β, IL-6 and TNF-α in ileum. (H) The immunofluorescence staining of lysozyme, scale bar: 100μm. (I) Mean number of Paneth cells per crypt. (J) Lysozyme+/DAPI+ quantification. (K) Western blot of Lysozyme. (L) Quantification of western blot results. The data are presented as the means ± SD. * P < 0.05 compared to N, # P < 0.05 compared to S, + P < 0.05 compared to T, & P < 0.05 compared to L. N, Normal group; S, Sham group; T, Severe traumatic brain injury group; L, Lysozyme treated group; NL: Normal + Lysozyme group; SL: Sham group + Lysozyme group. [file Image_2.tif]

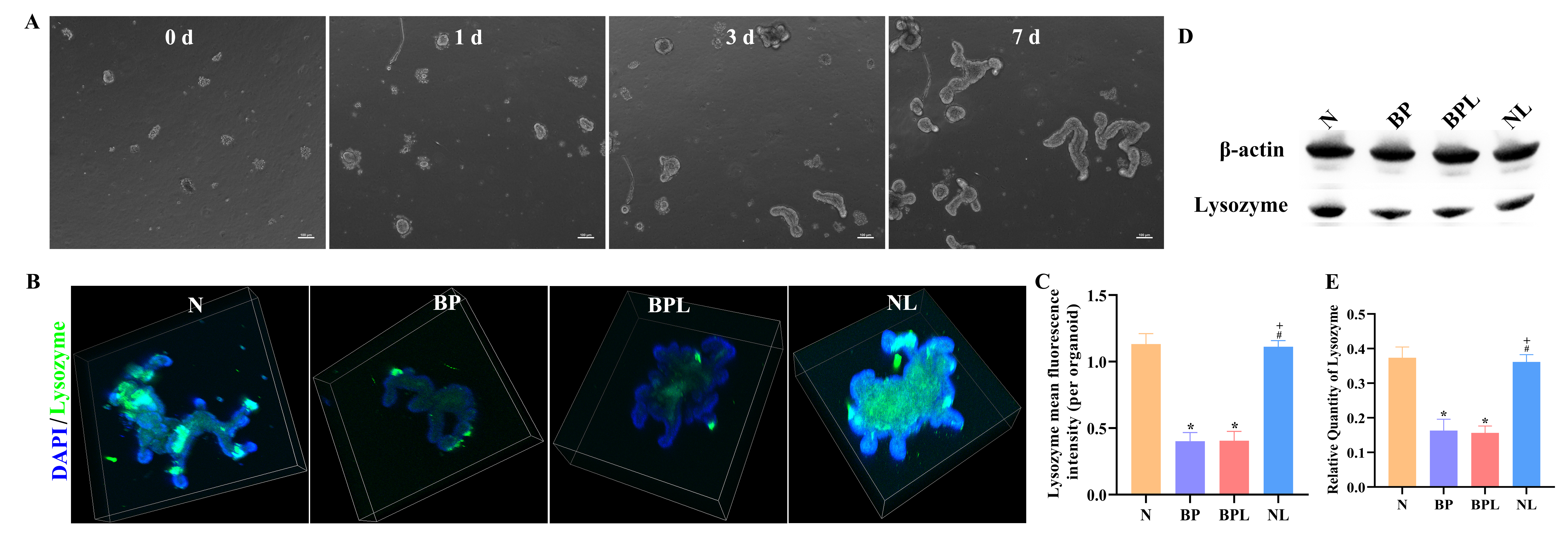

Supplement: Supplementary Figure 3 — Lysozyme had no effect on normal Intestinal organoids function. (A) Light microscopy photographs showing normal intestinal organoid, scale bar: 100μm. (B) The immunofluorescence staining of lysozyme in organoids. (C) The mean fluorescence intensity of lysozyme per organoid. (D, E) Western blotting and relative quantity of lysozyme. The data are presented as the means ± SD. * P < 0.05 compared to N, # P < 0.05 compared to BP, + P < 0.05 compared to BPL. N, Normal group; BP: Brain-derived proteins + Intestinal organoids co-culture; BPL, Brain-derived proteins + Lysozyme + Intestinal organoids co-culture. BP, Brain-derived proteins. [file Image_3.tif]
